# Reprints

**Published:** 1883-02

**Authors:** 


### REPRINTS.

We began in our January issue to publish a series of articles from the pen of Dr. P. P. Wells on dysentery, diarrhœa, rheumatism, etc. These are intended for binding into one volume when complete. The first eight pages appeared in January, but were not properly arranged. We therefore republish them this month with eight additional pages. The article of Dr. R. R. Gregg on "Overtaxed Brains," is also republished for similar reasons. This can be inserted in January issue in place of the former impressions.

